# The Importance of Recruitment of Low-Income Pregnant Persons in Interdisciplinary Research to Understand the Impact of Social and Environmental Determinants: Lessons Learned About Implementation and Recruitment

**DOI:** 10.1089/heq.2024.0112

**Published:** 2025-03-21

**Authors:** Erika Marquez, Amanda Haboush-Deloye, Elizabeth Lawrence, Selam Ayele, Courtney Coughenour, Nora Doyle, Wynona Dizon, Lizbeth Perez Rodriguez, Chloe Bombara

**Affiliations:** ^1^ Department of Environmental and Occupational Health, University of Nevada Las Vegas, Las Vegas, Nevada, USA.; ^2^ Nevada Institute for Children’s Research and Policy, University of Nevada Las Vegas, Las Vegas, Nevada, USA.; ^3^ Department of Sociology, University of Nevada Las Vegas, Las Vegas, Nevada, USA.; ^4^ Department of Obstetrics and Gynecology, UT Health, San Antonio, Texas, USA.; ^5^ Kirk Kerkorian School of Medicine, University of Nevada Las Vegas, Las Vegas, Nevada, USA.

**Keywords:** interdisciplinary research design, pesticide, built environment, vulnerable populations

## Abstract

**Background:**

Prior research indicates that enrolling underrepresented populations in clinical research is challenging. Although research has identified some barriers to participation and strategies to overcome them, studies have made little progress in being inclusive and representative. There remains a great need for including racial/ethnic minorities, low-income families, and pregnant women in research. The purpose of this article is to describe the implementation of enrollment strategies used in our study to understand the feasibility of building a maternal cohort and better understand the relationship between environmental and social impacts on maternal exposure and child outcomes.

**Methods:**

Working across multiple academic units, our team implemented equity-focused team science strategies to recruit diverse populations into a research study. The phases included development, conceptualization, implementation, and translation.

**Results:**

Our interdisciplinary team study used intentionality and commitment to deploy effective strategies including clearly defining the problem, selecting the correct team members to leverage expertise, clearly defining the study, establishing roles and responsibilities, representation, and clear and constant communication. A total of 100 pregnant women enrolled in our study using a team science interdisciplinary model.

**Discussion:**

Building interdisciplinary teams can help us understand complex problems, increase innovation, and develop effective solutions in policy and practice. More specifically, interdisciplinary teams can advance our ability to recruit diverse populations into research. Future studies should consider how to harness the strengths of the different research team members to achieve more inclusive participation.

**Health Equity Implications:**

This work has the potential to improve representation in research findings, enhance understanding of health disparities, and promote inclusive research practices.

## Introduction

Interdisciplinary research and inclusive approaches for recruiting diverse patient populations into research studies are critical pillars of scientific inquiry, fostering innovation and promoting equitable health outcomes. While there has been a great deal of emphasis on building interdisciplinary teams to address complex public health problems, the literature devotes little discussion to exploring how these teams function to support the equitable recruitment of vulnerable populations into research studies.

Ensuring the inclusivity and representation of the broader population is imperative. A recent report by the National Academies of Science^1^ highlights the consequences of the lack of diversity in clinical trials, including diminished generalizability, hindrance to innovation, and lack of access to effective interventions. A broad literature on the barriers and difficulties to recruiting underrepresented and excluded populations into scientific studies demonstrates several common, overlapping themes. The most common themes include a lack of trust in the scientific/medical community, potential participants’ resource needs, and cultural differences between the research team and the participants. Prior studies have also discussed strategies to combat and overcome these barriers, which include building trust with participants, incentivizing and making enrollment convenient, and delivering engagement with patients in a culturally competent way.^2–4^

Despite these efforts to improve recruitment for underrepresented and excluded populations, there has been a minor improvement in the inclusivity and representation of participants in clinical trials.^1^ For example, a recent report notes that 78% of participants in clinical drug trials from 2015 to 2019 were non-Hispanic White.^5^ The low rates of clinical trials including pregnant women are particularly concerning, as the Task Force on Research Specific to Pregnant Women and Lactating Women notes: “Research in pregnant women should be prioritized as the scientific need is large and research in this population is underrepresented.”^6^

As we continue to experience growth in populations and urban development, the role of social and environmental factors and understanding their connection to health and health outcomes becomes more critical. The built environment—encompassing housing, transportation systems, green spaces, infrastructure, and urban design—profoundly influences health through direct and indirect pathways.^7^ For pregnant persons, these influences are particularly significant, as the environments in which they live, work, and access health care can shape both maternal and fetal outcomes.^8^ Ethically, we must ensure that research outcomes are applicable to diverse populations. Moreover, we should ensure that the safety and efficacy of interventions and/or effects of drugs are consistent across demographic groups. The more diverse pregnant persons participate in studies, the more researchers are able to understand the impacts of the built environment on maternal-fetal health both in the short and the long term.

Interdisciplinary research teams have the potential to be on the frontier of science to address the impacts of the built environment among our increasingly diverse and often under-resourced populations. To guide this work, this article employs an interdisciplinary conceptual framework composed of four critical phases: development, conceptualization, implementation, and translation.^9^ By structuring the research process within this phased framework, this article seeks to describe the benefits and complexities of implementing interdisciplinary research studies while successfully enrolling diverse, low-income, pregnant persons in hopes that future studies can learn from our experiences. By linking findings and recommendations from prior studies to our on-the-ground efforts, we aim to provide a blueprint for study enrollment that could work in other organizational and geographic locations. We conclude with the importance of intentionality and commitment to effective communication, strong relationships, clear procedures, and principles of equity.

## Methods

### Description of a larger study: Measuring Pesticide Exposure in Pregnant Persons (MPEPP)

The Measuring Pesticide Exposure in Pregnant Persons (MPEPP) study aims to assess the prevalence of maternal stress and pesticides in the urine of low-income pregnant persons from communities historically marginalized by racism, xenophobia, and stratification. The main purpose of this study was to collect preliminary data to establish precedence for conducting a larger longitudinal study examining child outcomes related to exposure to pesticides *in utero*. A non-probability sampling strategy was used to recruit patients over a period of 15 months, from March 2022 to June 2023. Patients were enrolled at two university-affiliated OB/GYN clinics that provide inpatient and outpatient obstetric care. All participants were pregnant persons aged 18 or older seeking care at one of these clinics and were willing to complete the survey and study procedures.

The MPEPP study protocol was approved by the University of Nevada, Las Vegas (UNLV) Office of Research Integrity (approval #1631579-3).

### Results

Using an interdisciplinary conceptual model,^10^ we provide a case study describing the successes and challenges within each of the phases—development, conceptualization, implementation, and translation. Table 1 provides key recommendations during each of the phases identified by Tebes and Thai^10^ to support successful interdisciplinary teams and the recruitment of diverse populations.

**Table 1.** Considerations to Support Interdisciplinary Work and Recruitment of Diverse Populations, by Team Science Phase

Team science phases	Considerations for interdisciplinary teams	Equity considerations for recruitment of diverse populations
Development phase	Clearly define the problem Identify partners who have an institutional commitment to interdisciplinary work Determine the expertise needed Assess resources (e.g., funding, supplies, space, personnel) needed to execute the study Establish a team Develop a conceptual model	A commitment to health disparities research to uncover the root causes of health inequities A commitment to power sharing among the research team Establish partnerships with community organizations that serve diverse populations Incorporate diverse perspectives to ensure inclusivity
Conceptualization phase	Define the research purpose, objectives, and research questions Establish methods of communication among the team Establish clear roles and responsibilities for all team members Consider the capacity and competing responsibilities of the team and individual members Create an exhaustive list of all items needed to implement the study Create a workflow of the study execution detailing the order of activities and who is responsible Create a data analysis plan Plan for methods of dissemination	Ensure the development of interventions, surveys, questionnaires, and other study materials take into consideration language, culture, and local context Ensure research questions are relevant to the target population Cultural competency training for staff
Implementation phase	Disseminate study information to clinic staff who are not part of the study development team Identify advocates, such as other researchers, administrators, and medical personnel, to support your work Schedule regular meetings to assess progress and any implementation challenges Flexible modes of communication with research staff	Research team members that reflect the traits of the study population Availability of materials in languages needed Ensure flexibility within protocols to accommodate unique challenges (e.g., appointments) Active recruitment Assurance to participants that their time and participation are appreciated
Translation phase	Conduct data analysis Work with community partners on the best ways to disseminate and in what formats for each audience type	Translate findings into action (e.g., advancing science, improving the environment, informing policy, improving practices, and/or improving systems) Disseminate information in diverse formats Ensure information is relevant, relatable, and considers language (e.g., readability and languages spoken)

#### Development phase

##### Defining the problem

Defining the problem is a critical first step in conducting research on the social determinants of health and their role in the built environment and human health. Housing is one of those indicators and has an influence on both physical and emotional well-being through factors such as structural quality, location, and environmental safety. For instance, substandard housing conditions, including poor ventilation and proximity to agricultural fields, can increase pesticide exposure, which is known to have harmful effects on maternal stress, health, and fetal development.^11^ Pesticides not only pose direct toxicological risks but also exacerbate existing social and environmental inequities, disproportionately affecting low-income and marginalized communities where housing conditions may be suboptimal.^12^

Addressing these intertwined pathways requires an interdisciplinary approach. Housing conditions and pesticide exposure are not isolated issues; they intersect across disciplines; thus, an interdisciplinary research team allows for a comprehensive exploration of these complex dynamics. We wanted to assess outcomes of undue exposure to women and children at a local level—specifically, the implications of pesticide exposure to maternal stress, health, and fetal development.

##### Establishment of an interdisciplinary team

The study emerged from an interdisciplinary collaboration with faculty and students in the UNLV School of Public Health, Department of Sociology, and School of Medicine, which we refer to as the “research team.” Each member’s unique disciplinary perspective shaped the conceptualization of the study, the formation of the research question, and the overall direction of the study. The research team hypothesized that involving a diverse set of partners was critical to the success of the development of the study design, including determining study measures, data collection methodology, analysis, and recruitment of populations that were often under-resourced and represented in research.^13^ The relationship within the interdisciplinary research team was built through regular communication, mutual respect for each discipline’s contributions, power sharing, and a shared vision to address complex health disparities, all of which helped foster trust and collaboration among team members. For example, team members from each discipline actively contributed to decision-making processes, ensuring that all perspectives were valued and integrated into the study design. The Kirk Kerkorian School of Medicine at UNLV–specifically, its OB/GYN clinics—was essential to the study’s success. The clinics provided access to a population of pregnant individuals, including those from under-resourced backgrounds. The clinic director provided full support for the research effort.

#### Conceptualization phase

##### Study design

During the study design phase, the research team finalized the study purpose, objectives, and research questions through a collaborative process. This interdisciplinary approach sets the study apart by creating a more comprehensive and nuanced design. Public health experts emphasized community-level determinants and disparities, sociologists highlighted historical and contemporary structures and social norms influencing participation and outcomes, and medical professionals ensured clinical relevance and feasibility. These perspectives shaped a survey tool that captured environmental exposures, psychosocial stressors, and clinical health outcomes while remaining culturally sensitive to the target population.

##### Roles and responsibilities

The team members from Public Health and Sociology were responsible for securing resources (e.g., equipment, sampling supplies) and developing the study protocol and materials, with the UNLV School of Public Health providing the financial resources to purchase supplies and analyze the biological samples. The clinic director reviewed the research protocols, provided clinical expertise, and directed the clinic staff support to execute data collection. The clinic staff, including the office manager, medical residents, medical students, and medical assistants (MAs), assisted with the development of the workflow to guide implementation to ensure it had minimal impact on day-to-day clinic functions and did not add too much additional burden on the staff. While research staff wanted to be on-site to assist with recruitment, COVID-19 protocols were still in place when the institutional review board approved the study, and only essential personnel were allowed in the clinics. Therefore, explaining the study, consenting, and administering the survey were the responsibilities of the clinic staff. In addition, MAs were responsible for collecting urine samples, transferring the samples into appropriately labeled cryovials, and then storing the samples per Centers for Disease Control and Prevention (CDC) guidelines.

##### Communication

Regular meetings with clinic staff were vital to review and modify the workflow for recruitment, consent, survey collection, and urine sample collection procedures. The clinic staff were able to lead these discussions and voice the process that would work best for them, and during the study, the team met to determine if changes were needed. Regular meetings before and during the study’s implementation were critical in identifying and overcoming any logistical challenges. For instance, the study required the collection of urine samples, which is a normal part of an OB-GYN office visit. However, storing samples at the required temperatures to adhere to CDC analysis protocols is not a routine procedure. In addition, the clinics had limited space for new refrigerators. These logistical challenges were resolved by purchasing miniature refrigerators for each clinic so urine samples could be stored until they could be transported off-site by research staff to a freezer at the UNLV for storage until shipment to the CDC occurred once all samples were collected.

##### Study materials

All team members provided input on the development of an English and Spanish recruitment flyer, consent form, and study questionnaire. To maximize enrollment in the study, the team discussed how to present the informed consent in a clear, nonthreatening manner that would not shame parents if they did not meet the inclusion criteria, since shame might discourage them from participating in future research or engaging in health care services. Materials were shared with community partners to ensure cultural appropriateness, reading level, and understandable language. The recruitment flyer was used to promote the research study in the lobby to get patients familiar with the study before being asked by a medical assistant to participate. We designed the flyer to highlight the purpose of the study in simple terms and clearly list the eligibility criteria for the study. The questionnaire included questions about sociodemographic characteristics, housing, pesticide use and exposure, and maternal stress. Other materials needed included urine sample vials, de-identified patient labels for the vials, and the aforementioned freezer. To ease the burden on the clinic staff, team members from schools of public health and sociology prepared these materials and delivered them to the clinics.

#### Implementation phase

##### Representation

The clinics serve an ethnically diverse population of patients, many of whom speak Spanish as their first or only language. While the research team is ethnically diverse, the research staff supporting recruitment did not speak Spanish. An essential part of the research team’s collaboration with clinic staff was that a number of the clinic staff were fluent in Spanish. When a Spanish-speaking participant was identified, MAs who spoke Spanish and who were certified in human subjects research assisted with the recruitment, consent, and answering any questions.

##### Building collaboration

A few of the MAs and other clinic staff, such as front desk administrators, who were present during the planning meetings, were informed about the research study and recruitment process. However, it became immensely important to build support and buy-in among all clinic staff to support recruitment into the study. There was a lack of awareness about the research study among other physicians and clinic personnel not directly involved in the project. To improve recruitment efforts, the clinic staff who participated in developing the workflow shared the research study with all attendees at regular staff meetings, which helped increase awareness of the study among all clinic staff.

##### Clinic capacity

The study was developed during the COVID-19 pandemic. Thus, initial procedures required physicians, residents, and MAs to recruit study participants, limiting unnecessary personnel at the clinics. After several weeks of data collection, clinic staff only collected a few samples. The capacity of the clinic staff to be involved in the recruitment process did not go as anticipated. Resident physicians rotated in and out of the clinic frequently, and each of the available MAs worked with their designated physician on a tight schedule to ensure patients were served on time. Since it was a research site, other active studies were also conducting recruitment. A combination of all these factors resulted in a significant lag in recruitment. Fortunately, COVID-19 restrictions began to lessen, and research staff members were allowed to participate in the recruitment process, which resulted in a sharp increase in participation.

##### Communication

While diverse interdisciplinary teams are encouraged to conduct research, it can also come with challenges. One of the challenges we encountered was the need to maintain constant communication with the clinic staff, especially the MAs, to receive study updates. In order to address some of the communication barriers, we first identified a clinic contact that would be the primary point of contact at each of the clinics. This helped support consistent and constant communication between the team. The primary contacts took on a leadership role in the execution of the recruitment process and were well-informed about the study. They were critical in continuing to inform the research team about days to recruit based on the number of scheduled appointments, providing updates, and sharing concerns and challenges.

In addition, finding the best mode of communication was crucial. Initially, email was used as the primary method of communication, which worked well during the development phase of the study. Still, email was not the most effective method during implementation. To ensure a timely and constant exchange of information, the clinic and research staff agreed to use text messaging, which proved to be very effective and convenient in maintaining constant and timely communication. Text messaging included scheduling research staff to come to the clinic for recruitment or urine sample pickups or to communicate any challenges.

#### Translation phase

##### Recruitment outcomes

The collaborative research team efforts enrolled 100 pregnant women (Table 2). In addition, the demographic characteristics of these participants indicate that the team was able to recruit a diverse sample of pregnant women who are less likely to enroll in research studies. The ages of the participants ranged from 18 to 39, with a mean age of 27.7 (standard deviation = 6.1). The sample population was predominantly Hispanic/Latinx (51%), with other respondents identifying as White (13%), Black (12%), and Mexican American/Chicana (11%). Due to the small numbers of Asian and Pacific Islanders, we pooled these respondents with those marked “Other,” which totaled 9% of the sample. Roughly two-thirds of the sample completed the survey in English, with the remainder using the Spanish version. The sample is socioeconomically disadvantaged, with about one-third of respondents without a high school diploma or equivalent and more than half using public insurance such as Medicaid or Nevada Check-Up.

**Table 2.** Descriptive Characteristics of Enrolled Pregnant Women

	*N* (%)
Age	
18–22	25 (26%)
23–29	37 (38%)
30–39	35 (36%)
Race/ethnicity	
Black/African American	12 (12%)
Mexican American/Chicano	11 (11%)
Hispanic/Latinx	51 (51%)
White	13 (13%)
All other	9 (9%)
Survey language	
English	68 (68%)
Spanish	32 (32%)
Educational attainment	
Less than high school	31 (31%)
High school or equivalent	35 (35%)
Some college	19 (19%)
College degree or more	15 (15%)
Insurance status	
Public	51 (52%)
Private	17 (17%)
All other	30 (31%)
Weeks pregnant	
7–12	3 (3%)
13–24	27 (28%)
25–36	60 (63%)
37–39	5 (5%)
Previous live births	
0	34 (35%)
1	34 (35%)
2+	30 (31%)

## Discussion

This article aimed to describe the process and lessons learned during the recruitment of a diverse group of pregnant persons into a research study. Building interdisciplinary teams can help us understand complex problems, increase innovation, and develop effective solutions in policy and practice. More specifically, it can advance our ability to recruit diverse populations into research studies, enabling us to grow our knowledge of the built environment’s impact on maternal-fetal health, such as how housing quality, location, and access to goods and services affect maternal outcomes. This case study shows how building collaboration, flexibility, intentionality, and rapport into our research study design can increase the much-needed participation of diverse populations in research studies.

A team science framework can help lay foundational components for a research study. Such frameworks allow us to extend beyond discipline-specific approaches, fostering innovation and accelerating solutions to complex societal and scientific challenges through collaborative knowledge creation and practical application.^9^ The approach used in the case study is consistent with successful components identified in the existing literature, such as having a diverse team to enrich learning and the potential knowledge gained from research.^13^ Similar to participatory research, our interdisciplinary work required strong relationships for success.^14^ Building relationships included creating trust and sharing power in the decision-making process among the core team and the clinical staff directly supporting the research efforts. Lastly, establishing communication strategies, ensuring appropriate resources to execute the study were available, and establishing procedures all contributed to reaching study goals.^15^

There is a plethora of team science frameworks in the literature. However, few integrate equity as a core value within each phase’s considerations to mend the gap in diverse representation in research studies and translation of findings. Without this integration, participants exhibit a lack of trust. While participants in the current study did not voice a lack of mistrust, the legacy left by several studies, including the Tuskegee syphilis experiment and its abuse that resulted from structural racism, leaves today’s individuals, particularly those identifying as racial/ethnic minorities, wary of the medical establishment and researchers’ intentions.^3,16,17^ People are fearful of being used as “guinea pigs” and are mistrustful,^18^ which is particularly salient for pregnant persons who are not only thinking of their own safety but that of their children as well.

As we build interdisciplinary teams, we must also incorporate values of equity, such as fairness, justice, and inclusivity, to improve representation in studies and ensure that our findings develop interventions that impact and represent those most in need. To combat mistrust, researchers can build trust through various methods, such as incorporating participatory approaches into our study designs in which the study population helps develop and implement the study.^10,18^ Building trust can also include trusted providers introducing the study to potential participants^19^ before they interact with unfamiliar researchers, establishing rapport between researchers and participants, and creating connections with participants by having them repeatedly interact with the same member of the research team.^17,19,20^ Other important characteristics include attitudes of staff, flexibility in scheduling appointments, respect for the participant’s time, assurance that their participation is appreciated,^21^ frequent communication,^18^ and culturally sensitive practices.^19^

### Limitations

Despite the strengths of this study, there are limitations. Our recommendations and strategies regarding recruitment and retention may not generalize to studies with significantly larger sample sizes. In addition, our study worked with pregnant persons who were low-income, Spanish-speaking, and lived in urban areas. The results may differ from studies whose participants belong to other groups. Another limitation related to the participants was that we did not have a member of the focus group as part of the research team, mainly because the COVID-19 pandemic made such participation impossible. It would have been ideal to have a pregnant parent from one of the partner clinics or a similar clinic as part of the research team, as their perspective would have been invaluable. Lastly, as mentioned previously, this study utilized a collaboration with clinics associated with a university. Recruiting participants from clinics or other medical settings may show unique challenges that are not covered in this article.

## Conclusion

The intersection of interdisciplinary research and recruiting diverse populations into studies has far-reaching implications. As we better understand the mediators, moderators, and causal mechanisms between the built environment and health, we will be more equipped to translate science into action.^22^ Interdisciplinary research can offer substantial benefits, but aligning inherent differences in disciplinary perspectives, methodologies, and terminologies can be challenging. Navigating these challenges demands commitment and flexibility, which can be accomplished by implementing frameworks that facilitate effective collaboration while acknowledging and respecting the unique contributions of each discipline. Moreover, a team that adopts principles of equity within the development and execution of their research activities allows us to develop more effective interventions, policies, and practices that support community health. Our experience showed that while challenges to recruiting minority populations into clinical trials remain, steps can be taken to overcome these challenges. Future studies should diversify participant demographics in clinical studies to fill the gaps in existing literature regarding minority populations.

## Abbreviations Used

CDC: Centers for Disease Control and Prevention
MAs: Medical assistants
MPEPP: Measuring Pesticide Exposure in Pregnant Persons
UNLV: University of Nevada, Las Vegas
